# Human papillomavirus type 16 E6 and NFX1-123 mislocalize immune signaling proteins and downregulate immune gene expression in keratinocytes

**DOI:** 10.1371/journal.pone.0187514

**Published:** 2017-11-08

**Authors:** Justine Levan, Portia A. Vliet-Gregg, Kristin L. Robinson, Rachel A. Katzenellenbogen

**Affiliations:** 1 Center for Global Infectious Disease Research, Seattle Children’s Research Institute, Seattle, Washington, United States of America; 2 Pathobiology Interdisciplinary Program, Department of Global Health, University of Washington, Seattle, Washington, United States of America; 3 Department of Pediatrics, Division of Adolescent Medicine, University of Washington, Seattle, Washington, United States of America; Georgetown University, UNITED STATES

## Abstract

Human papillomavirus (HPV) is the most prevalent sexually transmitted infection, affecting an estimated 11% of the world’s population. The high-risk HPV types (HR HPV) account for approximately 5% of the global burden of cancer and thus cause high morbidity and mortality. Although it is known that persistent infection with HR HPV is the greatest risk factor for developing HPV-associated cancer, and that the HPV early proteins E6 and E7 dysregulate immune detection by its host cells, the mechanisms of immune evasion by HR HPV are not well understood. Previous work in the laboratory identified the endogenous cytoplasmic host protein NFX1-123 as a binding partner of the HR HPV type 16 oncoprotein E6 (16E6). Together NFX1-123 and 16E6 affect cellular growth, differentiation, and immortalization genes and pathways. In a whole genome microarray, human foreskin keratinocytes (HFKs) stably expressing 16E6 and overexpressing NFX1-123 showed a diverse set of innate immune genes downregulated two-fold or more when compared to 16E6 cells with endogenous NFX1-123. We demonstrated that 16E6 and NFX1-123 decreased expression of pro-inflammatory cytokines and interferon-stimulated genes (ISGs) in 16E6 HFKs at the mRNA and protein level. Knock down of NFX1-123 in 16E6 HFKs resulted in a derepression of innate immune genes, pointing to the requirement of NFX1-123 for immune regulation in the context of 16E6. Studies using immunofluorescent microscopy revealed that 16E6 and NFX1-123 disturbed the normal localization of signaling proteins involved in initiating the immune response. This study identifies NFX1-123 as a critical host protein partner through which 16E6 is able to subvert the immune response and in turn permit a long-lived HR HPV infection.

## Introduction

There are over 200 types of human papillomaviruses (HPV), which infect keratinocytes of the stratified squamous epithelium [1]. Those that specifically target mucosal epithelium are further categorized as low-risk HPV (LR HPV) or high-risk HPV (HR HPV) based on their epidemiologic association with cancer [2,3]. HR HPV cause nearly all cervical cancer, the fourth most common cancer in women, in addition to other anogenital and oropharyngeal cancers [2,4–6]. Altogether, cancers caused by HR HPV account for approximately 5% of the global burden of cancer [7]. The greatest risk factor for development of a HPV-associated cancer is a persistent infection with a HR HPV [8]. Although the immune response clears most of these infections, a persistent infection remains for some people [9–12]. The factors that contribute to a persistent HR HPV infection are not fully defined, but it is clear that effective avoidance of the host immune detection and response is integral to this process.

In addition to their structural role, keratinocytes are involved in immune system functioning. During the early stages of an HPV infection, the host innate immune response is the first line of defense against the infection. Multiple cells are involved in promoting an immune response against HPV infection, including dendritic cells, Langerhans cells, natural killer T cells, and keratinocytes. Keratinocytes provide robust immune defenses, expressing pathogen recognition receptors (PRRs) that activate signaling cascades and lead to production of soluble immune effector proteins such as cytokines and interferon-stimulated genes [13]. Despite these defenses, there can be a prolonged period between HPV infection and its clearance—months, even years [14–16]. This prolonged is a risk factor for development of oncogenic disease. The delay between infection and clearance indicates that HPV, and specifically HR HPVs, have evolved strategies to evade the innate immune responses of keratinocytes. Indeed, upon infection of keratinocytes, HR HPV actively subvert and hinder immune function. These disruptions are predominantly mediated by the HR E6 and E7 oncoproteins (reviewed in [17] and [18]).

Immune evasion mechanisms mediated by E6 and E7 generally act to prevent production of pro-inflammatory cytokines, interferons, and other antiviral genes or to disrupt antigen presentation [10]. E6 has been shown to dampen interferon signaling by binding and preventing activity of the signaling molecule Tyk2 and the transcription factor IRF3, both members of the interferon signaling pathway [19,20]. E6 also downregulates expression of individual cytokines IL-18 and MCP-1, and a surface protein important for recruitment of antigen presenting cells, E-cadherin [21–23]. E7 inhibits interferon signaling through inactivation of IRF-1, prevents antigen presentation through suppressing expression of MHC Class I family members, and inhibits recognition of cytoplasmic viral nucleic acid [24–26]. While it is evident that E6 and E7 predominate the immune evasion strategies of HPV, the specific molecular mechanisms and endogenous cellular protein partners that drive these immune deregulatory functions remain largely unknown. Protein partnerships are essential to any E6 and E7 functionality, as the HPV proteins have no enzymatic capacity of their own, and the protein partners utilized are typically host proteins [27–31].

Our laboratory has previously studied the partnership between the E6 protein of HR HPV type 16 (16E6) and the host cytoplasmic protein NFX1-123 [32–34]. NFX1-123 is endogenously expressed in human epithelial cells, and it is increased in cervical cancer cell lines [35]. NFX1-123 has been shown to bind 16E6 [32]. They together target cellular pathways important to the virus and malignant development such as cellular immortalization, growth, and differentiation. We have previously demonstrated that 16E6 and NFX1-123 increase Notch1, a master regulator of cell proliferation and differentiation, and increase hTERT, the catalytic subunit of telomerase [32–36]. These studies highlight how the partnership between 16E6 and NFX1-123 modulates cellular genes critical to promoting an ideal environment for HPV infection, as HPV requires its host cells to both grow and differentiate and HPV-associated cancers universally activate telomerase to drive cellular immortalization. By dysregulating these genes and pathways, 16E6 and NFX1-123 may aid in establishing and supporting a long-term HPV infection and potential malignancy.

We were interested in exploring what other pathways are manipulated by 16E6 and NFX1-123 to alter the host cell and engender an environment supportive of persistent HR HPV infection. In this study, we establish that 16E6 and NFX1-123 deregulated the innate immune response of keratinocytes, impeding the normal expression of diverse immune genes including cytokines and interferon stimulated genes. The data presented here identified NFX1-123 as a critical host protein partner through which 16E6 was able to subvert the immune response, revealing another cellular process targeted by this protein partnership that could further promote a HR HPV infection.

## Materials and methods

### Plasmids

The plasmids for FLAG-tagged NFX1-123WT, pBabe-puro 16E6, LXSN vector control, scramble short hairpin control, and short hairpin 1 RNA to NFX1-123 (sh1) have been described previously [32].

### Tissue culture

Primary cultures of human keratinocyte cells (HFKs) were established as previously described from neonatal foreskins [37]. HFKs were grown in EpiLife medium (Life Technologies, Carlsbad, CA) supplemented with 60uM calcium chloride, penicillin-streptomycin, and human keratinocyte growth supplement (Life Technologies, Carlsbad, CA). 293T cells were obtained from ATCC (CRL-3216) grown in Dulbeccos’ Modified Eagle Medium (DMEM) supplemented with 10% fetal bovine serum and penicillin-streptomycin.

### Microarray

The microarray was performed and analyzed as previously described [36]. Briefly, total RNA was isolated using TRIzol reagent (Life Technologies, Carlsbad, CA), purified using the RNeasy kit (Qiagen, Valencia, CA) according to manufacturer’s instructions, and converted to cDNA. cDNA were labeled and hybridized to the HumanHT-12 v4 Expression BeadChip array (Illumina, San Diego, CA). Data are accessible in the Gene Expression Omnibus database under accession number GSE43082. Array data were analyzed using GeneSpring GX11.5.1 (Agilent Technologies, Santa Clara, CA).

### Retrovirus production and transduction of primary keratinocytes

Retrovirus was produced in 293T cells by a previously described transient vesicular stomatitis virus G-pseudotyped virus (VSV-G) production protocol [38] or by a previously described lentiviral production protocol [39]. Briefly, pBABE-puro or LXSN retroviral constructs were cotransfected with VSV-G, tat, and pJK3 into 293T cells using FuGENE6 (Roche, Alameda, CA); short hairpin NFX1-123 (sh1) or scramble (scr) c-FUGW constructs were cotransfected with VSV-G and delta 8.9 plasmids into 293T cells as above. Following infection, retrovirus was serially collected, concentrated by ultracentrifugation, mixed with Polybrene (8 mcg/ml) (EMD Millipore, Billerica, MA), and incubated with 50 to 60% confluent HFKs. Three hours later, the Epilife medium was replaced. For FN123, LXSN vector control, and pBabe-puro 16E6 infections, the cells were expanded 24 hours post-transduction, and placed under neomycin/G418 selection (50 mcg/ml) or puromycin selection (0.5 mcg/ml) 48 hours post-transduction. All lentivirus infections (scramble and sh1) were confirmed by green fluorescent protein expression.

### Western blot analysis

Whole-cell protein extracts were resolved by SDS-polyacrylamide gel electrophoresis and transferred to polyvinylidene difluoride (PVDF; Millipore, Billerica, MA). Blots were probed with anti-NFX1-123 (1:1000), anti-p53 (1:1000, Calbiochem, San Diego, CA), (anti-TRAF6 (1:500; Cell Signaling, Danvers, MA), anti-TRIF (1:1000, Abcam, Cambridge, MA), anti-TAK1 (1:500, Abcam, Cambridge, MA), anti-TAB1 (1:500, Abcam, Cambridge, MA), anti-TAB2 (1:500, Abcam, Cambridge, MA), and anti-GAPDH (1:100,000; Abcam, Cambridge, MA) primary antibodies. The secondary antibodies used were anti-mouse IgG HRP (1:10,000; Cell Signaling, Danvers, MA) or anti-rabbit IgG HRP (1:5,000, Cell Signaling, Danvers, MA). The rabbit polyclonal anti-NFX1-123 antibody was generously provided by Dr. Ann Roman. Animals were immunized with peptide composed of amino acids 1102–1120 of NFX1-123. All films were scanned using Epson Perfection V700 and imported using Adobe Photoshop.

### RNA expression analysis and quantitative real-time PCR

Total RNA was isolated with TRIzol reagent (Life Technologies, Carlsbad, CA). Total RNA (1 mcg) was DNase treated and used to generate cDNA with random hexamer primers and SuperScript IV reverse transcriptase (Life Technoloiges, Carlsbad, CA) as described previously [32,35,36]. Quantitative real-time PCR (qPCR) was performed using an ABI StepOne Plus system (Applied Biosystems, Foster City, CA). Amplification was carried out using TaqMan master mix and the following pre-designed Taqman probes: GAPDH (4333764F), CXCL1 (Hs00236937_m1), TNF (Hs00174128_m1), OAS1 (Hs00196324_m1), and OAS2 (Hs00942643_m1) according to the manufacturer’s instructions (Applied Biosystems, Foster City, CA). Reactions were performed in triplicate. Data analysis was performed using the Pfaffl method [40] to determine relative expression levels, with GAPDH to normalize mRNA levels within each sample. Values graphed are the mean fold-change in each sample compared to control, and error bars graphed represent 95% confidence intervals for the technical replicates (n = 3).

Primer sequences for NFX1-123, and 36B4 were described previously [32,41] Amplification for primer based qPCR was carried out as previously published using Power SYBR Green Master Mix (Life Technologies, Foster City, CA) [32]. Reactions were performed in triplicate, and NFX1-123 was normalized to the housekeeping gene 36B4. Values graphed are the mean fold change in each sample compared to control, and error bars using NFX1-123 and 36B4 primers represent 95% confidence intervals for the technical replicates (n = 3).

### Immunofluorescent staining and microscopy

Immunofluorescence was performed on cells grown on cover slips (#1 1/2 x 18mm). Cells were fixed with 4% paraformaldehyde for fifteen minutes at room temperature, washed with PBS and permeabilized with ice cold methanol/acetone (1:1) for 30 seconds. After blocking with PBS containing 1% TWEEN-20 and 3% BSA for one hour at room temperature, cover slips were incubated overnight at four degrees Celsius in blocking buffer with the same primary antibodies used in Western blot analysis above (Abcam, Cambridge, MA), unless otherwise stated: anti-TRAF6 (1:500), anti-TRIF (1:500), anti-TAK1 (1:500), anti-TAB1 (1:500), and anti-TAB2 (1:500). All secondary antibodies were obtained from Life Technologies (Carlsbad, CA) and were as follows: anti-rabbit AlexaFluor 488 (1:500), anti-mouse AlexaFluor 546 (1:500), and anti-goat AlexaFluor 633 (1:500). Secondary antibodies were incubated in the dark for one hour at room temperature with Hoechst stain (1:5000, Thermo, Waltham, MA). Cover slips were washed with cold PBS and mounted in glass slides with ProLong reagent (Thermo, Waltham, MA). Confocal images (stacks) were acquired at 0.2 micron spacing with an Olympus 60x oil immersion objective as specified in the figure legends with an Applied Precision DeltaVision RT microscope system (Applied Precision, Issaquah, WA). The exposure times were kept constant for each fluorescence channel within each experiment and antibody used. Stacks were deconvolved using a constrained iterative algorithm with DeltaVision SoftWoRx program, version 4.1.2.

### High-content analysis of immunofluorescent microscopy

#### Total intensity

Previously deconvolved and normalized images of HFK, 16E6/control, and 16E6/FN123 cells grown on coverslips, fixed and co-stained for either TAB1, TAB2 and TAK1 or TRIF, TRAF6 and TAK1 were quantified using the FIJI scientific imaging analysis platform [42]. The fluorescent channels were split into greyscale images and analyzed for mean average intensity. A minimum of 30 cells from each of three separate biological backgrounds were used for quantification.

#### Subcellular localization

Previously deconvolved and normalized images of HFK, 16E6/control, and 16E6/FN123 cells cells grown on coverslips, fixed and co-stained for TAB1, TAB2 and TAK1 or TRIF, TRAF6 and TAK1 were split by channels into greyscale images. These were visually sorted according to whether the staining was above background, and whether the staining was in the cytoplasm or had coalesced into distinctive brighter staining perinuclear or nuclear foci. The Hoechst nuclear staining was used as a general mask. A minimum of 60 cells from each of three separate biological backgrounds were used for quantification.

## Results

### Microarray and pathway analyses of genes upregulated in HFKs expressing 16E6 and overexpressing NFX1-123

**Fig 1:**
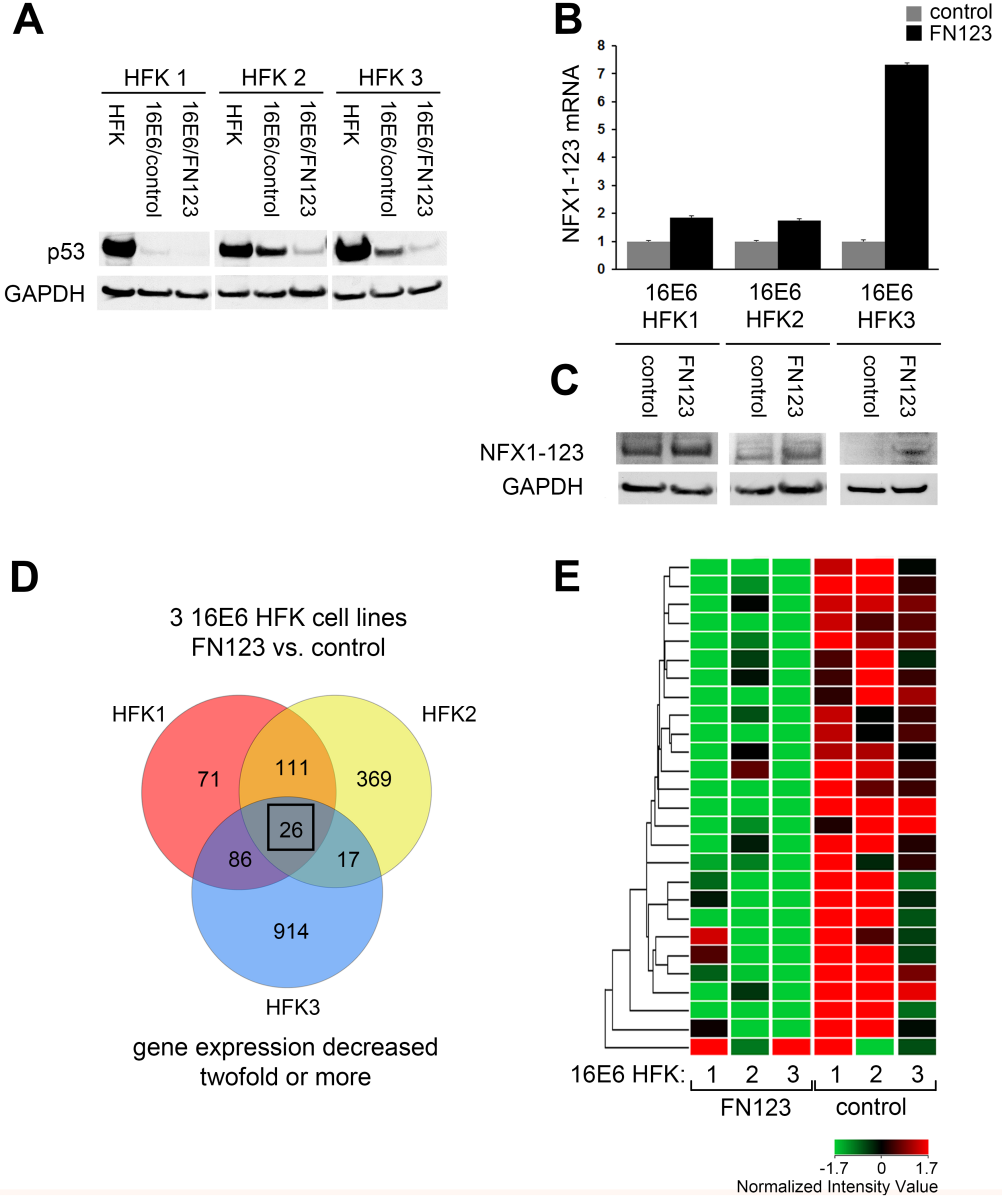
Microarray analysis of genes downregulated in 16E6 HFKs with increased NFX1-123. Whole genome expression microarrays were conducted in HFKs stably expressing 16E6 and overexpressing NFX1-123 or with endogenous levels of NFX1-123. (A) Three biologically independent HFKs were transduced with 16E6 and p53 protein levels assessed. (B) 16E6 HFK lines 1, 2, and 3 were transduced with NFX1-123 overexpression construct (FN123) or vector control (control). Levels of NFX1-123 mRNA and protein expression levels were quantified and compared. All qPCRs were normalized to the housekeeping gene 36B4, and all error bars represent 95% confidence intervals from the technical replicates shown (n = 3). GAPDH = loading control for (A and C). (D) Venn diagram of genes whose average expression was decreased at least two-fold in 16E6/FN123 cells compared to 16E6/control. Box indicates 26 genes represented in (D) (E) Heat map with hierarchical clustering of the 26 genes decreased in 16E6/FN123 cells compared to 16E6/control.

NFX1-123 is a cytoplasmic protein whose partnership with 16E6 has previously been shown to co-regulate expression of genes such as Notch1, epithelial cell differentiation markers, and hTERT [32–36]. To explore what other transcripts and cellular processes might be altered by this partnership, we conducted a gene expression microarray in keratinocytes that stably expressed 16E6 and overexpressed NFX1-123. First, three biologically independent human foreskin keratinocyte cell lines (HFKs 1–3) were stably transduced with 16E6. We confirmed protein expression and function of 16E6 through Western blot of p53, as 16E6 targets p53 for degradation (Fig 1A). For each HFK cell line, there was a significant decrease in p53 protein in cells transduced with 16E6 compared to their isogenic, non-transduced control (Fig 1A, 16E6/scr and 16E6/FN123 versus HFK). These 16E6 HFKs (1–3) were then expanded and transduced a second time with either a FLAG-tagged NFX1-123 construct (FN123) or with a scramble short hairpin RNA (control). The scramble short hairpin RNA does not target any known genes by BLAST search, and HFKs transduced with this scramble shRNA display levels of NFX1-123 equal to endogenous levels. Quantitative real-time PCR (qPCR) analysis showed that for each independent 16E6 HFK cell line, there was nearly a two-fold increase in NFX1-123 mRNA expression in FN123 cells versus control (Fig 1B) that was also reflected at the protein level (Fig 1C).

An Illumina beadchip microarray was performed in the three independent biologic backgrounds for each 16E6/control and 16E6/FN123 (GEO Accession number GSE43082). There were over 200 genes identified whose average expression was decreased by at least two-fold in at least two out of the three independent 16E6/FN123 HFK lines compared to 16E6/control HFKs. There were 26 genes found to be downregulated in all three cell lines (Fig 1D, box). These 26 genes are represented in a heat map with unsupervised hierarchical clustering (Fig 1E).

**Fig 2:**
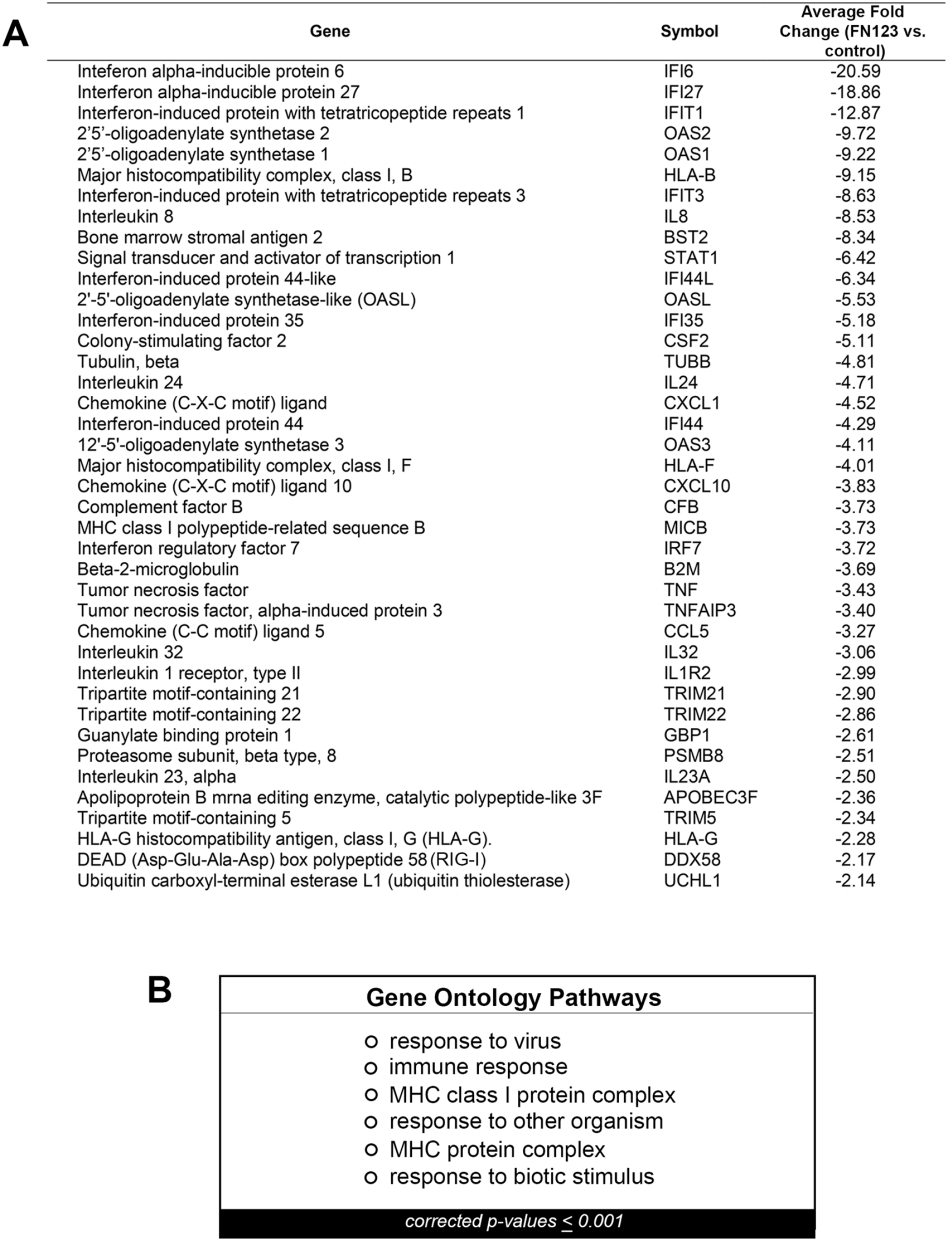
Genes and pathways decreased in 16E6/FN123 HFKs. (A) A subset of the over 200 genes that were decreased in 16E6/FN123 cells compared to 16E6/control. Fold change shown is the average fold change over all 16E6 HFK cell lines in which that gene was decreased. (B) Microarray data were analyzed using GeneSpring GX11.5.1, and the significant Gene Ontology pathways for the collection of genes decreased in two out of three 16E6 HFK cell lines were found. Only pathways with a p-value ≤ 0.001 are shown.

A subset of the over 200 genes whose average expression was decreased in at least two of three HFK cell lines are listed in Fig 2A. These include proinflammatory cytokines such as IL-8 and IL-23, structural elements such as tubulin, and a proteasome complex protein, PSMB8. Given the large number of genes that were decreased in 16E6 HFKs with increased NFX1-123 expression, we were interested in determining whether there were common nodes or pathways targeted for downregulation. Pathways that were enriched for in these downregulated genes were examined via Gene Ontology analyses, and the results are shown in Fig 2B. P-values were adjusted for multiple comparisons and only those pathways with a p-value ≤0.001 are shown. Pathways critical to viral clearance and immune detection were returned as highly enriched. Even with these stringent conditions of analyses, a number of different, yet related pathways involved in the immune response were identified. Crucially, in a parallel microarray comparing HFKs that overexpressed NFX1-123 or had endogenous levels of NFX1-123 but without 16E6 co-expression, none of these same immune pathways were found to be enriched. Thus, it was only in the context of 16E6 that NFX1-123 was involved in regulating the immune response.

### Overexpression of NFX1-123 decreased innate immune gene expression in 16E6 HFKs

**Fig 3:**
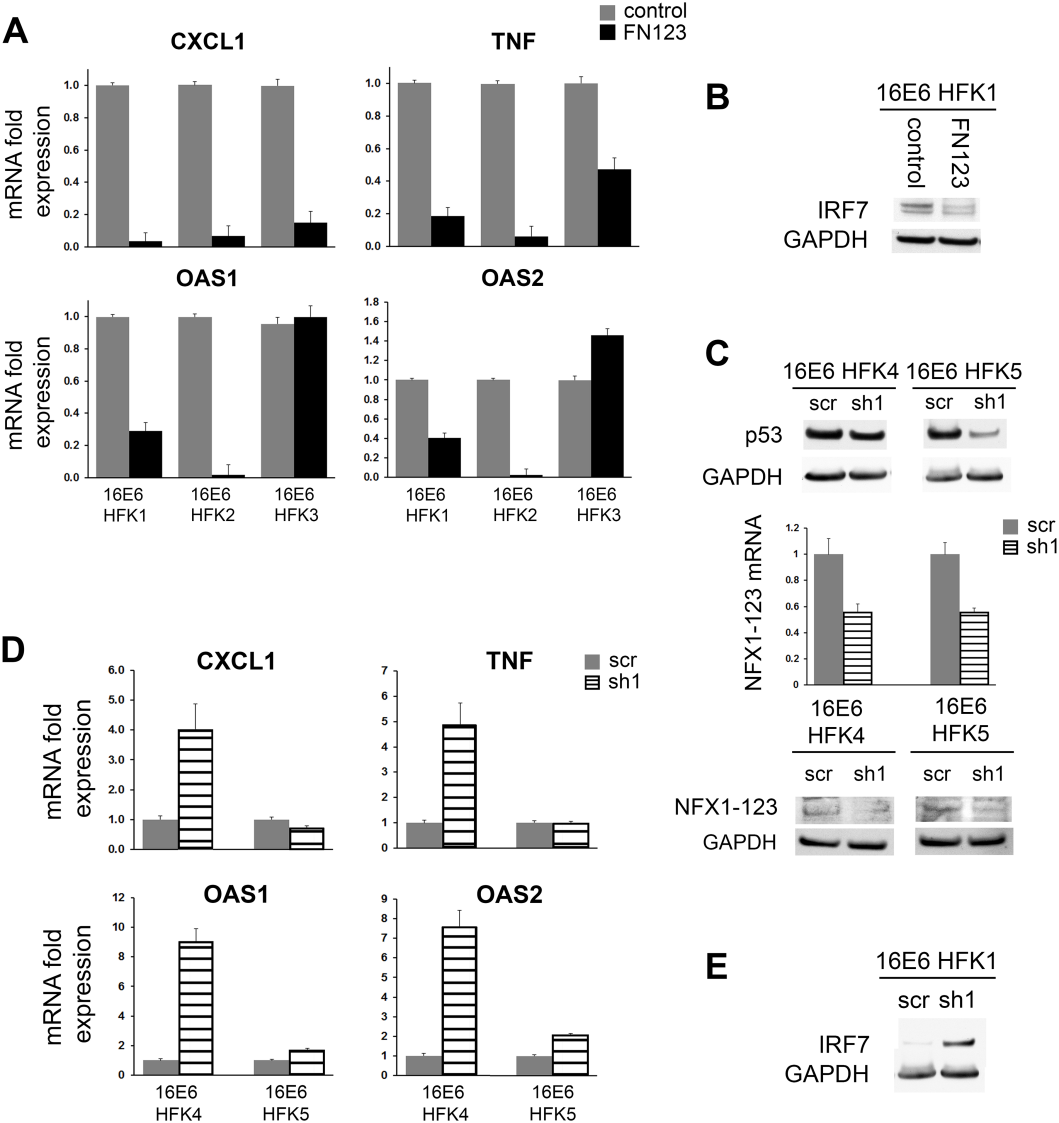
Immune gene expression decreased in 16E6 HFKs with more NFX1-123 or increased in 16E6 HFKs with NFX1-123 knockdown. Expression levels of innate immune genes were examined in 16E6 HFKs with increased NFX1-123 compared to endogenous levels, or with NFX1-123 decreased via short hairpin RNA. (A) mRNA levels of CXCL1, TNF, OAS1, and OAS2 were quantified by qPCR in 16E6/FN123 cells compared to 16E6/control. 16E6 HFKs with increased NFX1-123 showed a decrease in many innate immune genes. (B) Protein levels of IRF7 in 16E6/control cells or 16E6/NFX1-123 cells. 16E6 HFKs overexpressing NFX1-123 had reduced IRF7 protein. (C) Two biologically independent HFKs were transduced with 16E6 and p53 protein levels assessed. Cells were then transduced with a scramble short hairpin construct (scr) or a short hairpin RNA targeting NFX1-123 overexpression construct (sh1). Levels of NFX1-123 mRNA and protein expression levels were quantified and compared. (D) mRNA levels of CXCL1, TNF, OAS1, and OAS2 were quantified by qPCR in 16E6/sh1 cells compared to 16E6/scr. 16E6 HFKs with decreased NFX1-123 showed a rebound in many innate immune genes. (E) Protein levels of IRF7 in 16E6/scr or 16E6/sh1 cells were assessed. 16E6 HFKs with NFX1-123 knocked down had increased IRF7 protein. Innate immune gene qPCRs were normalized to the housekeeping gene GAPDH, while NFX1-123 qPCRs were normalized to the housekeeping gene 36B4. All error bars represent 95% confidence intervals from the technical replicates shown (n = 3) GAPDH = loading control.

Following our microarray studies, we further validated the roles of 16E6 and NFX1-123 in co-regulating genes of the immune response at the mRNA and protein levels. We returned to the three 16E6 HFK cell lines in which the microarrays were completed (16E6 HFK 1, 2, and 3) for validation. Fold changes in mRNA expression were quantified by qPCR for a set of genes that were decreased in the screening microarray of 16E6/FN123 cells (Fig 3A). Although there were differences based on biologic background, greater NFX1-123 expression in 16E6 HFKs resulted in reduced mRNA expression of the innate immune genes CXCL1, TNF, OAS1, and OAS2 when compared to 16E6/control HFKs with endogenous amounts of NFX1-123 (Fig 3A). In all three independent 16E6 HFK lines, CXCL1 had at least a ten-fold decrease in mRNA expression, while TNF had at least a 2.5-fold decrease. Mirroring results from the microarray, OAS1 and OAS2 were decreased in two out of three 16E6 HFK cell lines by at least 2.5-fold. Modulation of innate immune factors at the protein level was also seen. Compared to 16E6/control cells, FN123 cells of 16E6 HFK1 had lowered amounts of IRF7 protein, a transcription factor critical to the interferon response (Fig 3B).

### Knock down of NFX1-123 led to rebound of innate immune gene expression in 16E6 HFKs

Having found that a diverse set of innate immune genes were downregulated in 16E6 HFKs with overexpression of NFX1-123, we then hypothesized that knock down of the endogenous amount of NFX1-123 would result in a derepression of these genes. Two additional biologically independent HFK lines were transduced with 16E6 (16E6 HFK 4 and 5), then expanded and transduced with either a short hairpin RNA targeting NFX1-123 (sh1) or a scramble short hairpin control (scr). Again, expression and function of 16E6 were confirmed by p53 Western blot (Fig 3C). NFX1-123 expression was quantified five days post transduction. In both 16E6 HFK 4 and 16E6 HFK 5, sh1 reduced NFX1-123 mRNA by approximately 50%. NFX1-123 protein was also reduced (Fig 3C). The mRNA expression of CXCL1, TNF, OAS1, and OAS2 was subsequently assessed. In 16E6 HFK 4, both CXCL1 and TNF levels rebounded four- to nearly five-fold when NFX1-123 was decreased compared 16E6/control cells, although there was little to no shift in these two genes in 16E6 HFK 5 (Fig 3D). Again, this reflects differences based on biological background of the primary HFKs. Both 16E6 HFK 4 and 16E6 HFK 5 had significant increases in OAS1 and OAS2 upon knock down of NFX1-123. Despite differences in the magnitude of derepression, the interferon-stimulated OAS genes were consistently increased two- to nine-fold in sh1 cells compared to scr (Fig 3D). Similarly, decreasing NFX1-123 expression resulted in an increase in the level of IRF7 protein (Fig 3E).

### NFX1-123 overexpression did not globally decrease levels of innate immune signaling proteins

**Fig 4:**
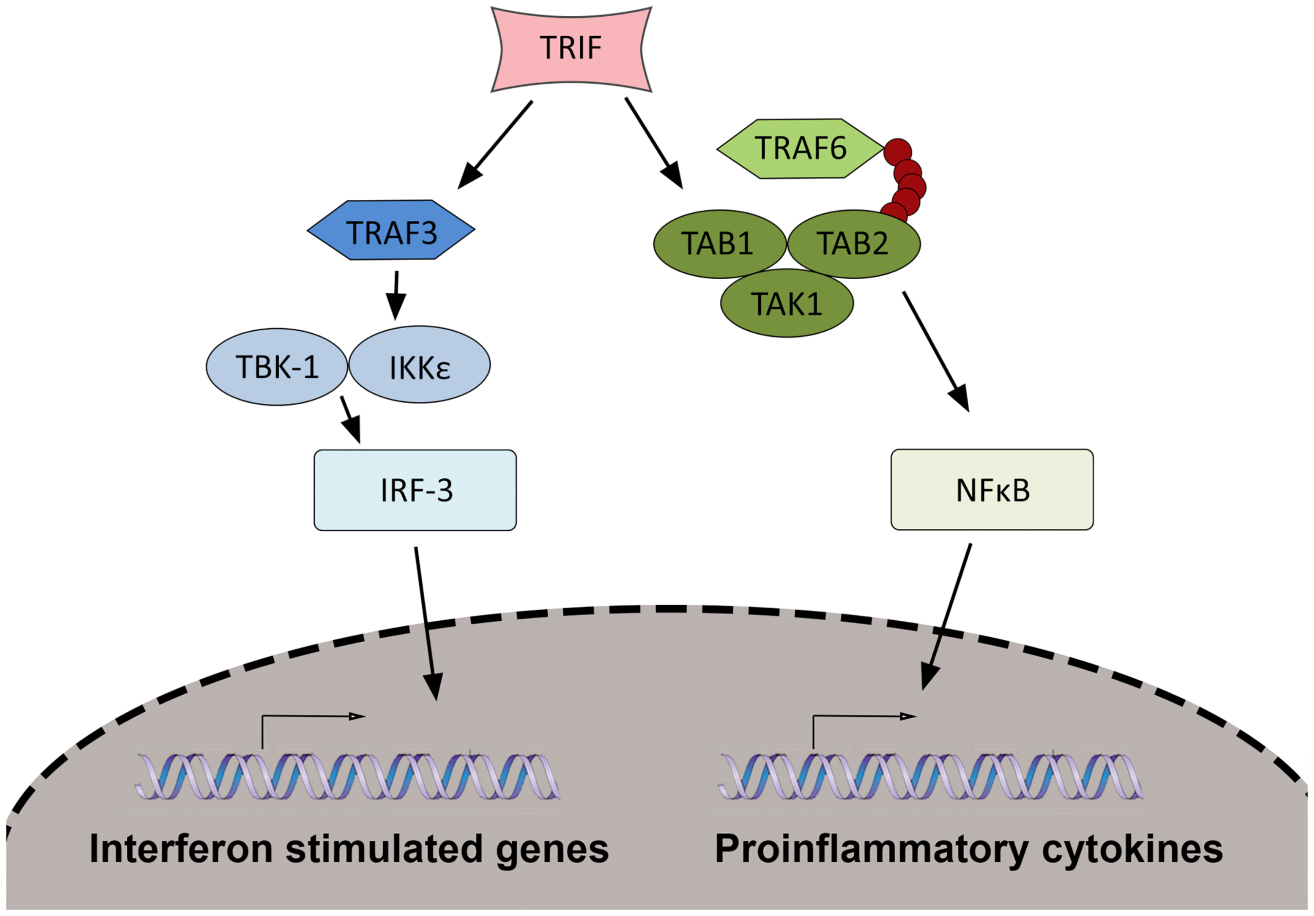
Innate immune signal transduction pathways in keratinocytes. An example of an immune signaling cascade downstream of pattern recognition receptors is shown. Stimulus from pathogen infection is sensed by PRRs, communicated through the adaptor protein TRIF, and further communicated through complexes formed by signaling proteins shown. These result in translocation of the transcription factors IRF-3 or NFkB to induce expression of interferon-stimulated genes, or proinflammatory cytokines and other mediators.

In normal keratinocytes, the expression of genes such as IL-8, CXCL1, and other genes found to be downregulated in our screening microarray are induced through immune signaling pathways. Input, in the form of pathogen assault or stimulus, is communicated through these signaling pathways; output, in the form of proinflammatory cytokines, interferon-stimulated genes, and other factors, creates the innate immune response. A few common signaling pathways are responsible for induction of hundreds of these immune genes. The broad number of innate immune genes found modulated in the screening microarray and the lack of alignment with a singular category, such as proinflammatory cytokines, led us to hypothesize that the dysregulation of the immune response genes might be occurring at a level above, or prior to, the direct downregulation of the genes themselves. We therefore next queried whether co-regulation of the immune response by 16E6 and NFX1-123 occurred through disruption of the signaling pathways that govern expression of innate immune genes. A diagram of an immune signaling cascade and the key proteins involved is depicted in Fig 4.

**Fig 5:**
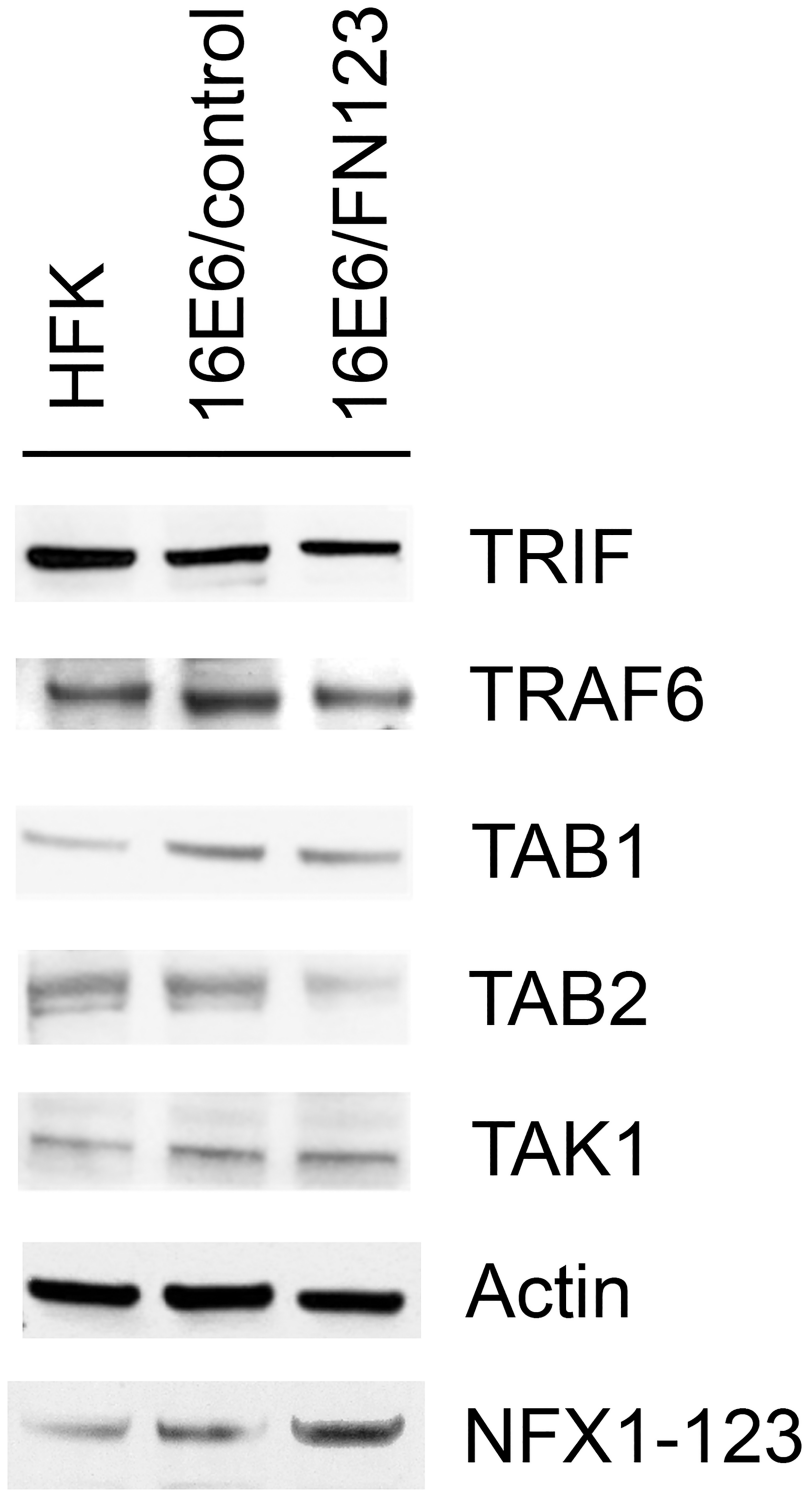
Total amounts of innate immune signaling pathway proteins are not globally decreased. Three independent HFK cell lines were serially transduced with 16E6 and then either FN123 or vector control. Whole cell lysates were collected from each, and subsequent protein blots were probed for the immune signaling proteins TRIF, TRAF6, TAK1, TAB1, and TAB2. NFX1-123 overexpression was confirmed in FN123 cells compared to HFK or 16E6/control. Actin = loading control. Data shown are from one representative cell line.

The mechanism by which 16E6 and NFX1-123 dysregulate protein signaling complexes could occur in a number of ways, including 1) a decrease in total signaling protein level, 2) sequestration of proteins, or 3) prevention of signaling complex formation. We addressed the first possibility by comparing total quantities of key signaling proteins in normal HFKs, 16E6 HFKs with endogenous levels of NFX1-123, and 16E6 HFKs with overexpressed NFX1-123. As before, HFKs were serially transduced with 16E6, and then either a FLAG-tagged NFX1-123 construct (FN123) or a vector control (control). Whole cell lysates were collected from each, and subsequent protein blots were probed for the immune signaling proteins TRIF, TRAF6, TAK1, TAB1, and TAB2. Experiments were conducted in three independent HFK cell lines with representative data of one cell line shown here (Fig 5). Interestingly, there appeared to be largely no difference in total quantities of these proteins across normal HFK, 16E6/control, or 16E6/FN123 cells. There was a slight decrease in levels of TAB2 in 16E6/FN123 cells compared to 16E6/control, but no difference between normal HFKs and 16E6/control cells, nor any significant differences for any of the other signaling proteins examined (Fig 5). Thus, altering total levels of upstream signaling proteins does not appear to be a mechanism by which 16E6 and NFX1-123 blunt the immune response.

### Subcellular localization of innate immune signaling proteins was altered with increased NFX1-123

**Fig 6:**
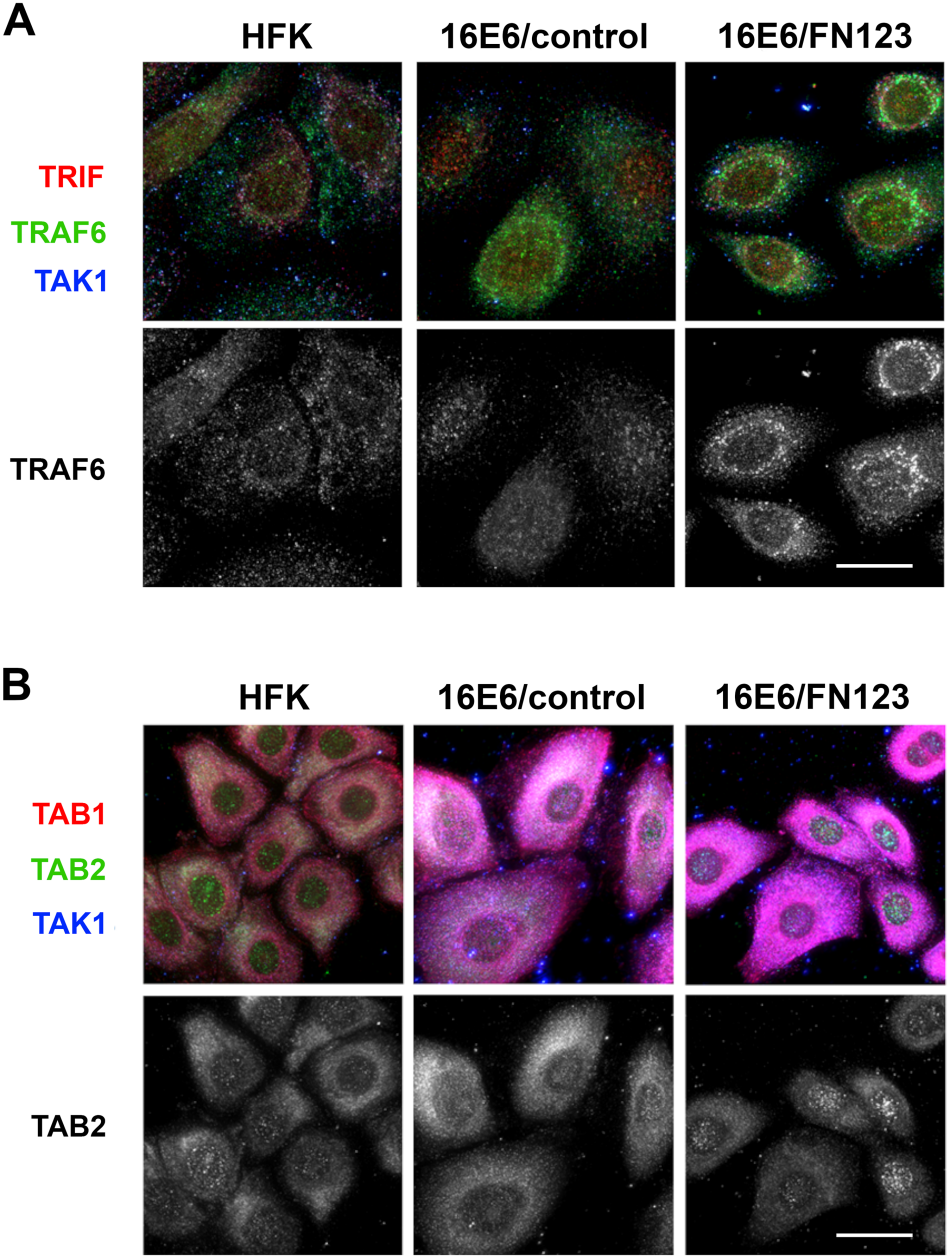
Co-localization and subcellular localization of innate immune signaling pathway proteins are altered by 16E6 and NFX1-123. Immunofluorescence was performed on HFKs, 16E6/control HFKs, and 16E6/FN123 HFKs grown on cover slips. Cells were fixed, permeabilized, and probed with primary antibodies. Scale bars = 20 micron. (A) Cells were stained for TRIF (red), TRAF6 (green), and TAK1 (blue) and shown as a merged image (top panel). The green channel representing TRAF6 was isolated and displayed as a greyscale image (bottom panel). 16E6/FN123 cells show strong perinuclear localization of TRAF6 compared to HFKs or 16E6/HFKs. (B) Cells were stained for TAB1 (red), TAB2 (green), and TAK1 (blue) and shown as a merged image (top panel). The green channel representing TAB2 was isolated and displayed as a greyscale image (bottom panel). 16E6/FN123 cells contain bright, punctate staining of TAB2 in the nuclear compared to 16E6/control cells.

Ordered formation of signaling complexes is essential for full functioning of the signaling pathways depicted in Fig 4. Often, an event that occurs in one complex results in activation and allows for formation of the next. Sequestration of signaling proteins, and preventing their participation in signaling complexes, would therefore inhibit signaling and induction of immune genes. We employed immunofluorescent microscopy and high-content analysis of images to examine whether 16E6 and NFX1-123 disrupt the subcellular localization of single proteins or co-localization of multiple proteins. Normal HFKs, 16E6/control HFKs, and 16E6/FN123 HFKs were fixed, permeabilized, and stained for the signaling proteins listed in Fig 5 as two groups: TRIF/TRAF6/TAK1 (Fig 6A) and TAK1/TAB1/TAB2 (Fig 6B). These two groupings represent two signaling complexes whose formation after an immune stimulus is required for completion of the signaling cascade (depicted in Fig 4) and subsequent induction of target genes.

The subcellular localization of TRAF6 appeared to change dramatically with overexpression of NFX1-123 (Fig 6A, bottom row, 16E6/FN123). Whereas staining of TRAF6 appears diffuse throughout the cytoplasm in normal HFKs and 16E6/control HFKs, increased expression of NFX1-123 in the presence of 16E6 resulted in an altered pattern. With more NFX1-123, TRAF6 appeared to migrate to distinct, perinuclear foci. Additionally, co-localization of TRIF and TRAF6 seemed to increase with expression of 16E6 and even more with NFX1-123 overexpression (Fig 6A, top row).

Similar to TRAF6, subcellular localization of TAB2 also appeared to be altered with more NFX1-123 (Fig 6B, bottom row). In 16E6/FN123 cells, TAB2 staining gained the appearance of bright, punctate foci that were localized to the nucleus. Although it is not clear from these data what impact this would have upon TAB2 functioning, it is clear that the normal localization of this important signaling protein is disturbed. Although the total expression levels of these signaling proteins may not be significantly changed, there were alterations to the subcellular localization and co-localization of these proteins. Appropriate localization of these signaling proteins, at the appropriate time, is crucial to their functioning and to subsequent induction of the innate immune response.

Quantitative data was also obtained from the immunofluorescent images. First, the average intensity of staining, representing total amount of innate signaling proteins (Table 1), was determined in HFKs, 16E6/control cells, and 16E6/FN123 cells. Images from three independent biologic backgrounds were analyzed and mean fluorescent intensity normalized to HFK values. For TRIF, there was not a significant difference in intensity of staining across the three cell types, indicating that there was not a substantial difference in the total amount of protein present. There was a relative increase in expression of TAK1 and TAB1 that occurred when 16E6 was expressed (16E6/control vs HFK), but with greater expression of NFX1-123, the levels of these proteins were blunted and returned to typical levels seen in normal HFKs. Interestingly, there was a significant decrease in staining intensity of TRAF6 in 16E6/FN123 cells compared to either HFKs or 16E6/control, mirroring the results seen in Western blot analyses. There was a 30% to 40% reduction in intensity of staining for TRAF6 in 16E6/FN123 cells compared to HFK or 16E6/control. Similarly, there was also a striking decrease in TAB2 fluorescence in 16E6/FN123 cells compared to HFK and 16E6/control cells, with approximately a 40% reduction in staining intensity.

**Table 1:** Whole cell mean fluorescent intensity of innate immune signaling proteins.

	HFK	16E6/control	16E6/FN123
**TRIF**	100 (99.5–100.5)	116.4 (115.9–116.9)	85.8 (85.4–86.3)
**TRAF6**	**100 (99.5–100.4)**	**121.4 (120.8–121.9)**	**69.8 (69.4–70.2)** ^^,^ ^‡^
**TAK1**	100 (99.7–100.3)	159.2 (158.5–159.6) ^¤^	104.1 (103.8–104.5)^‡^
**TAB1**	100 (99.7–100.5)	190.3 (189.8–190.9) ^¤^	109.1 (108.7–109.4)^‡^
**TAB2**	**100 (99.7–100.5)**	**90.0 (89.7–90.4)**	**35.4 (35.2–35.6)** ^^,^ ^‡^

Mean fluorescent intensity of the innate immune signaling proteins displayed were normalized to the HFK and averaged across the three biologic cell line backgrounds. 95% confidence intervals are shown in parentheses. Statistical significance determined by one-way ANOVA with Bonferroni post-hoc test. All symbols shown indicate p-value < 0.05:

¤ = HFK compared to 16E6/control;

^ = HFK compared to 16E6/FN123;

^‡^ = 16E6/control compared to 16E6/FN123. Values for TRAF6 and TAB2 are significantly decreased in 16E6/FN123 cells compared to both HFK cells and 16E6/control cells and are shown in bold.

To quantify the shifts in location of TRAF6 and TAB2 that were seen in HFKs with 16E6 and overexpressed NFX1-123, subcellular localization of these proteins was also quantified (Table 2). HFK and 16E6/control cells had a similar ratio of nuclear/perinuclear to cytoplasmic TRAF6 foci, with 26–28% nuclear/perinuclear and 72–74% cytoplasmic. In 16E6/FN123 cells, however, this ratio was reversed, with the higher proportion of TRAF6 found to be nuclear/perinuclear (63%) rather than cytoplasmic (37%). Similarly, although TAB2 was predominately in the cytoplasm for HFK and 16E6/control cells (69–77%), it became highly perinuclear in 16E6/FN123 cells, with the cytoplasmic percentage falling to only 37% and the nuclear/perinuclear increasing to 63%. Thus, not only were total amounts of TRAF6 and TAB2 protein decreased in whole cell extracts and by immunofluorescent staining, their subcellular localization were also shifted in 16E6 HFKs overexpressing NFX1-123.

**Table 2:** Subcellular localization of innate immune signaling proteins TRAF6 and TAB2.

	HFK		16E6/control		16E6/FN123	
	Nuc/Peri	Cyto	Nuc/Peri	Cyto	Nuc/Peri	Cyto
**TRAF6**	26% (21–32%)	74% (68–79%)	28% (22–34%)	72% (66–78%)	63% (56–70%)	37% (30–44%)
**TAB2**	23% (17–28%)	77% (72–83%)	31% (25–37)%	69% (63–75%)	63% (57–68%)	37% (31–42%)

Subcellular localization of innate immune signaling proteins TRAF6 and TAB2 were determined and averaged across the three biologic cell line backgrounds. 95% confidence intervals are shown in parentheses.

## Discussion

Collectively, the work presented in this study establishes a novel role in immune evasion for the protein partners HPV type 16 E6 and NFX1-123. We show that together, 16E6 and NFX1-123 inhibit the typical expression of diverse immune genes in keratinocytes. Greater levels of NFX1-123 resulted in a decrease of a broad range of immune factors such as proinflammatory cytokines and interferon-stimulated genes. The wide diversity of immune functions in the downregulated genes suggests that this may be a broad effect. Crucially, this only occurred when 16E6 was present, revealing the essentiality of partnership between 16E6 and NFX1-123 for this regulation. Furthermore, we demonstrate that NFX1-123 is required for immune dysregulation by 16E6 (Fig 3). Knock down of NFX1-123 by half rescued baseline expression of innate immune genes. With less NFX1-123, the ability of 16E6 to decrease immune gene expression was diminished. Interestingly, 16E6 and NFX1-123 appeared to achieve this immune modulation by interfering with intracellular immune signaling, thereby preventing expression of innate immune genes and dampening the keratinocyte immune response.

The innate immune response is the first line of defense against pathogenic infection; it is therefore not surprising that HPV has evolved mechanisms that target numerous, overlapping points to dismantle these defenses and ensure a long-lived infection. Particularly important in the context of HPV infection is the etiological connection between a persistent infection with HR HPV types and development of anogenital and oropharyngeal cancers. The substantial clinical consequences of successful immune evasion by HR HPV make study of these mechanisms paramount. Both initial development of malignancy and overall progression of oncogenic disease are tied to whether or not there is a successful immune response. Identifying the specific ways in which HR HPV subvert this response may uncover possible points of intervention to circumvent persistent infection, decrease disease burden, and lower cancer incidence.

Our findings complement the many previously identified mechanisms by which 16E6 inhibits the immune response [19,22,43–45]. As indicated earlier, these strategies often overlap in their ultimate effects upon innate immunity—namely, inhibiting the induction of key effector genes such as proinflammatory cytokines, chemokines, and interferon-stimulated genes. It benefits the virus to have redundancy in these tactics, and thus our studies are a logical addition to the expansive repertoire defined by other groups. Universal to the varied immune evasion strategies employed by HPV is the dependence on and partnership with host proteins. Here, we identify NFX1-123 as a novel protein partner involved in innate immune evasion by 16E6. Notably, the host proteins through which HPV enacts its subversion of the immune response do not fall within a single category. Studies have identified interactions with DNA sensors [26,46], transcription factors [43,45], and enzymes involved in post-translational modification, such as UCHL1 and IFRD1 [47,48]. It is evident that the oncoproteins of HR HPV are able to hijack a vast array of host proteins to modify the cellular environment, regardless of the usual function of that endogenous protein. Previous work from our laboratory has demonstrated that 16E6 and NFX1-123 are involved in cellular growth, longevity, and differentiation [32–36]. We now define a new role for this partnership in immune evasion.

The data presented here also represent the first documentation of NFX1-123 driving a downregulation of gene expression. NFX1-123 has been previously shown to expression of two cellular genes important in HPV-associated cancers, hTERT and Notch1 [33,36]. NFX1-123 posttranscriptionally increases the expression of hTERT through RNA binding and stabilization. Notch1 is a master regulator of cell growth and differentiation across many cell types, and perturbations in Notch signaling can confer either oncogenic or tumor suppressor effects on a cell, depending on its context [49,50]. The new role for NFX1-123 as an inhibitor of immune gene expression suggests that in the context of 16E6, NFX1-123 can be classified more broadly as a gene regulator rather than simply an activator. Taken together, partnership between 16E6 and NFX1-123 changes the cell to engender an environment supportive of HPV infection, both by enhancing pathways important for cellular growth and inhibiting pathways that would lead to viral elimination.

Interestingly, the molecular mechanism employed by 16E6 and NFX1-123 in modulating the immune response is also novel for this particular partnership. Whereas NFX1-123 works together with cytoplasmic poly(A) binding proteins to increase hTERT expression at the posttranscriptional level [33], the effects upon gene expression here seem to be indirectly, through upstream signaling complexes. This is similar to known mechanisms of immune evasion mediated by HR HPV, which frequently target signaling crossroads to prevent the interferon response and the NFkB pathway. HPV 18 E6, for example, binds Tyk2, prevents phosphorylation of downstream kinases, and inhibits transcription of interferon-stimulated genes [19]. Multiple HR HPV types prevent the expression or function of STAT1, a signaling protein integral to the interferon signaling cascade [44,51,52]. Even amongst mechanisms that target signaling proteins, the specific molecular action by which HR HPV interferes with signaling can differ. Inhibition can be achieved by degradation of the proteins, physical sequestration, and altering post-translational modifications, amongst others. The data presented here indicate that 16E6 and NFX1-123 may employ the second option of the three, disrupting subcellular localization of immune signaling proteins to prevent normal formation of signal transduction complexes. For example, we documented an altered pattern of TRAF6 localization upon overexpression of NFX1-123 (Fig 6). TRAF6 migrated from primarily cytoplasmic to perinuclear foci, and this sequestration of TRAF6 likely decreased the probability of TRAF6 interfacing with protein partners. Additionally, co-localization between immune signaling proteins was disrupted, as seen in increased co-localization of TRAF6 and TRIF (Fig 6). Such abnormal, forced co-localization of proteins in the absence of immune stimulus may prevent these complexes from forming correctly when a stimulus is present. This would stop the signal cascade at its earliest steps and prevent the expression of immune genes.

Future research will further examine how 16E6 and NFX1-123 deregulate the immune response during an active stimulation. Keratinocytes constitutively express some cytokines, but are also are primed to respond quickly, producing soluble effector proteins to both initiate an anti-pathogenic (antiviral) state and recruit or activate other resident immune cells. This state is largely possible due to constant expression of signaling proteins that, upon stimulation, rapidly transduce a signal and induce gene expression. 16E6 and NFX1-123 appear to disturb the normal localization and likely function of the signaling proteins. We thus hypothesize that there are functional consequences upon the ability to mount an immune response both at the keratinocyte level and the larger epidermal level. Keratinocytes are the first-line responders to any infection and act as a link to the rest of the immune effector cells in the region [13]. 16E6 and NFX1-123 inhibiting immune signaling has implications not only for whether a viral infection is eliminated, but also whether a malignant growth is detected and cleared.

In conclusion, the studies presented here identify NFX1-12 as a host protein that is co-opted by 16E6 to deregulate the innate immune response of keratinocytes and inhibit the expression of immune genes. This is achieved by perturbing the normal localization of signaling proteins, resulting in a broad inhibition. These data support previous work that elucidates how 16E6 and NFX1-123 contribute to a cellular environment that supports long-lived HPV infection. Persistent infection with HR HPV is the highest risk factor for cancer development; uncovering the mechanisms of immune evasion by HR HPV could advance understanding of how to prevent persistent infection and ultimately cancer.

## Supporting information

S1 Fig Full Western blots of p53, NFX1-123, and GAPDH for 16E6 HFK 1–3. Protein levels of (A) p53, (B and C) NFX1-123, or (A and D) GAPDH were assessed in three biologically independent HFK backgrounds. Per biological background, samples were HFKs, HFKs transduced with 16E6 and vector control, or HFKs transduced with 16E6 and NFX1-123 overexpression construct.
(TIF)

S2 Fig Full Western blots of IRF7, p53, GAPDH, and NFX1-123 for 16E6 HFK 1, 16E6 HFK4, and 16E6 HFK 5. Protein levels of (A) IRF7 and(B) GAPDH were assessed in 16E6 HFK 1. Boxed lanes in (A) indicate HFK1 samples. HFKs were serially transduced with 16E6 and either scrambled short hairpin control or short hairpin RNA construct targeting NFX1-123. Protein levels of (C) p53, (D and G) NFX1-123, and (E and F) GAPDH were assessed in 16E6 HFK 4 and 16E6 HFK 5. HFKs were transduced with 16E6 and either scrambled short hairpin control or short hairpin RNA construct targeting NFX1-123. Protein levels of (H) IRF7 or (I) GAPDH were assessed in 16E6 HFK 1. HFKs were serially transduced with 16E6 and either vector control or NFX1-123 overexpression construct.
(TIF)

S3 Fig Full Western blots of TRAF6, TRIF, TAB1, TAB2, TAK1, Actin, and NFX1-123 for 16E6 HFK. Protein levels of (A) TRAF6, (B) TRIF, (C) TAB1 and TAB2, (D) TAK1,(E) Actin, and (F) NFX1-123 were assessed in HFK cells. Samples were HFKs, HFKs transduced with 16E6 and vector control, or HFKs transduced with 16E6 and NFX1-123 overexpression construct.
(TIF)
