# Supplementary figures and images for: Human papillomavirus type 16 E6 and NFX1-123 mislocalize immune signaling proteins and downregulate immune gene expression in keratinocytes
Source: PLoS One. 2017 Nov 8;12(11):e0187514. doi: 10.1371/journal.pone.0187514 (PMC5695606; doi:10.1371/journal.pone.0187514)

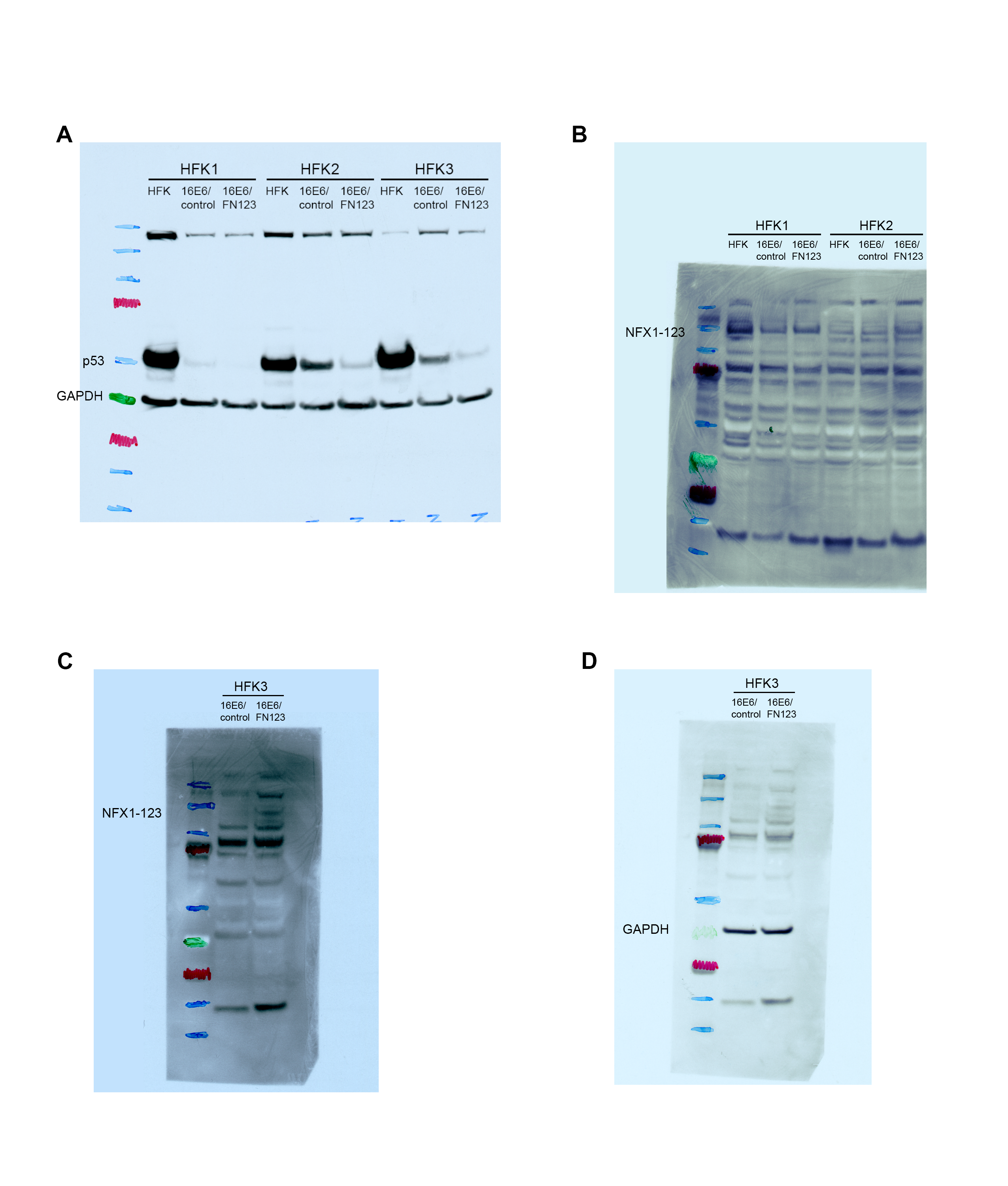

Supplement: S1 Fig — Protein levels of (A) p53, (B and C) NFX1-123, or (A and D) GAPDH were assessed in three biologically independent HFK backgrounds. Per biological background, samples were HFKs, HFKs transduced with 16E6 and vector control, or HFKs transduced with 16E6 and NFX1-123 overexpression construct. (TIF) [file pone.0187514.s001.tif]

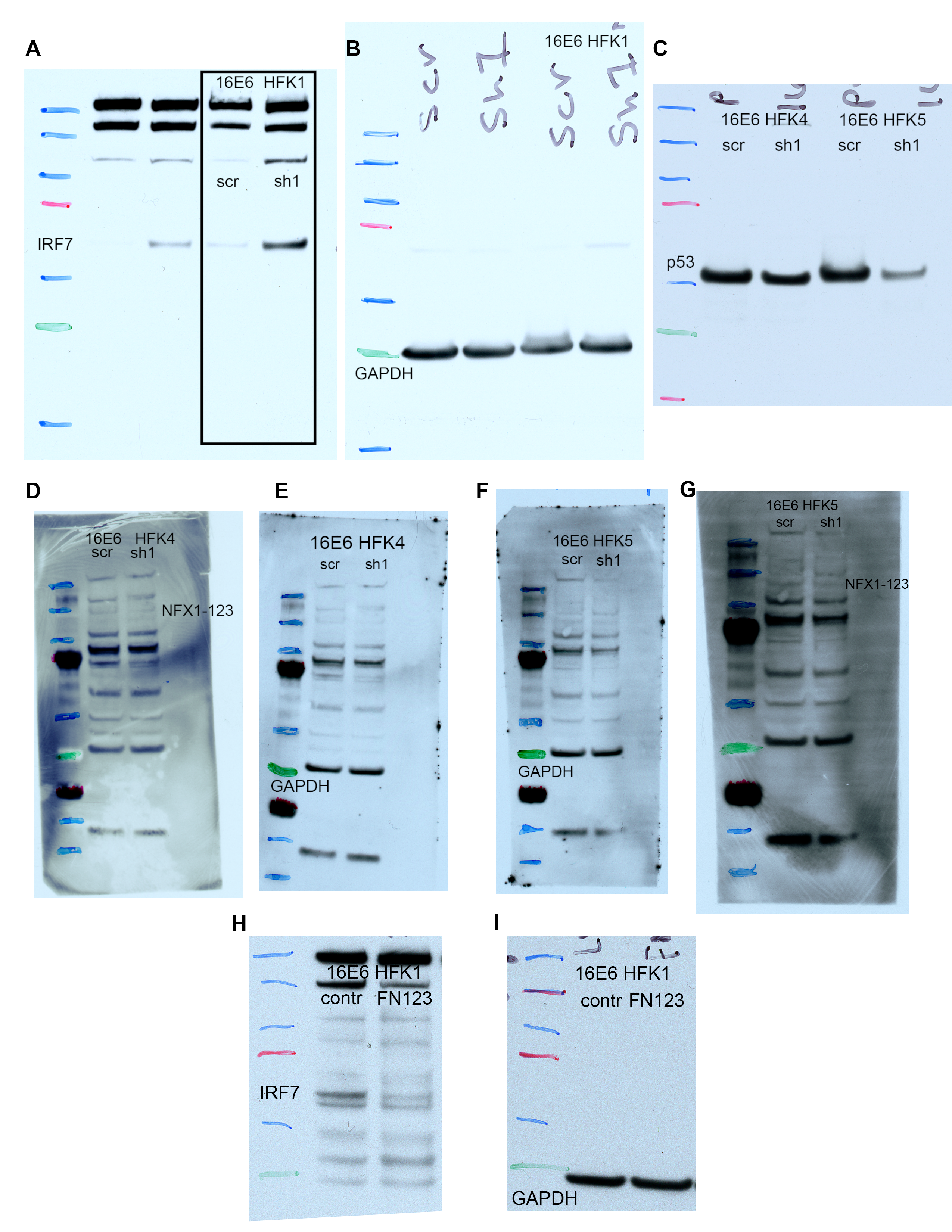

Supplement: S2 Fig — Protein levels of (A) IRF7 and(B) GAPDH were assessed in 16E6 HFK 1. Boxed lanes in (A) indicate HFK1 samples. HFKs were serially transduced with 16E6 and either scrambled short hairpin control or short hairpin RNA construct targeting NFX1-123. Protein levels of (C) p53, (D and G) NFX1-123, and (E and F) GAPDH were assessed in 16E6 HFK 4 and 16E6 HFK 5. HFKs were transduced with 16E6 and either scrambled short hairpin control or short hairpin RNA construct targeting NFX1-123. Protein levels of (H) IRF7 or (I) GAPDH were assessed in 16E6 HFK 1. HFKs were serially transduced with 16E6 and either vector control or NFX1-123 overexpression construct. (TIF) [file pone.0187514.s002.tif]

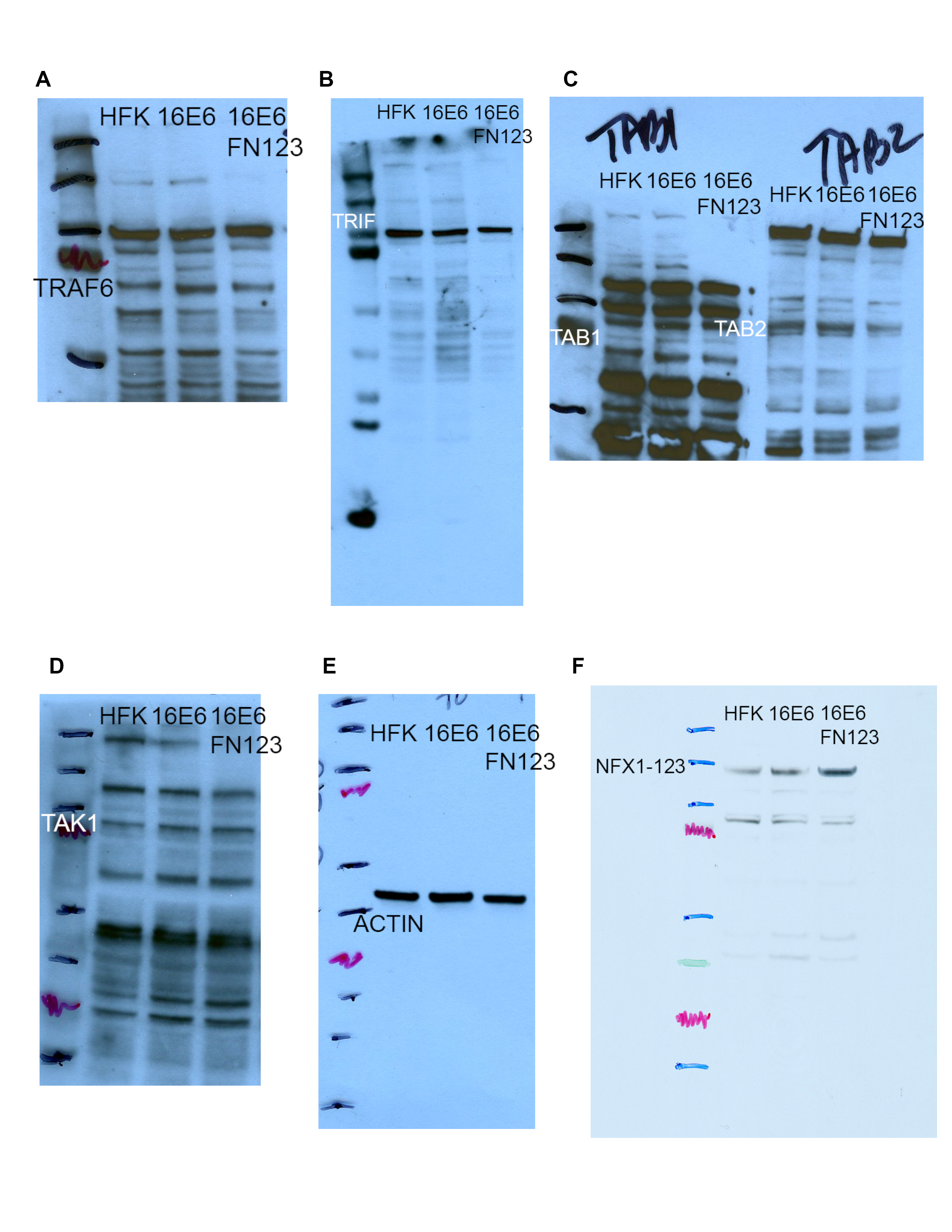

Supplement: S3 Fig — Protein levels of (A) TRAF6, (B) TRIF, (C) TAB1 and TAB2, (D) TAK1,(E) Actin, and (F) NFX1-123 were assessed in HFK cells. Samples were HFKs, HFKs transduced with 16E6 and vector control, or HFKs transduced with 16E6 and NFX1-123 overexpression construct. (TIF) [file pone.0187514.s003.tif]
